# Computational approaches for the reconstruction of optic nerve fibers along the visual pathway from medical images: a comprehensive review

**DOI:** 10.3389/fnins.2023.1191999

**Published:** 2023-05-26

**Authors:** Richu Jin, Yongning Cai, Shiyang Zhang, Ting Yang, Haibo Feng, Hongyang Jiang, Xiaoqing Zhang, Yan Hu, Jiang Liu

**Affiliations:** ^1^Research Institute of Trustworthy Autonomous Systems, Southern University of Science and Technology, Shenzhen, China; ^2^Department of Computer Science and Engineering, Southern University of Science and Technology, Shenzhen, China; ^3^Guangdong Provincial Key Laboratory of Brain-inspired Intelligent Computation, Department of Computer Science and Engineering, Southern University of Science and Technology, Shenzhen, China

**Keywords:** optic nerve fiber, visual pathway, image segmentation, fiber tracking, artificial intelligence, medical image analysis

## Abstract

Optic never fibers in the visual pathway play significant roles in vision formation. Damages of optic nerve fibers are biomarkers for the diagnosis of various ophthalmological and neurological diseases; also, there is a need to prevent the optic nerve fibers from getting damaged in neurosurgery and radiation therapy. Reconstruction of optic nerve fibers from medical images can facilitate all these clinical applications. Although many computational methods are developed for the reconstruction of optic nerve fibers, a comprehensive review of these methods is still lacking. This paper described both the two strategies for optic nerve fiber reconstruction applied in existing studies, i.e., image segmentation and fiber tracking. In comparison to image segmentation, fiber tracking can delineate more detailed structures of optic nerve fibers. For each strategy, both conventional and AI-based approaches were introduced, and the latter usually demonstrates better performance than the former. From the review, we concluded that AI-based methods are the trend for optic nerve fiber reconstruction and some new techniques like generative AI can help address the current challenges in optic nerve fiber reconstruction.

## 1. Introduction

The visual pathway is a general name for a series of brain tissues including the optic nerve (ON), optic chiasm (OC), optic tract (OT), lateral geniculate nucleus (LGN), optic radiation (OR), and visual cortex (VC) (Smith and Strottmann, 2001; Jäger, 2005). In the visual pathway, ON, OC and OT are formed by the axons of the retinal ganglion cells (Becker et al., 2010), while OR is formed by another type of optic nerve fibers. These two types of optic nerve fibers are connected at the LGN, which is a relay station of optic signals (Fujita et al., 2001). The optic never fibers along the visual pathway are responsible for the conduction of optic signals from the retina to the visual cortex and play significant roles in vision formation.

Optic nerve fibers can be affected by various ophthalmological diseases, e.g., glaucoma (Hernowo et al., 2011; Tellouck et al., 2016; Haykal et al., 2022), age-related macular degeneration (Prins et al., 2016; Yoshimine et al., 2018) and optic neuritis (Yamamoto et al., 2005; Spierer et al., 2010; Zhao et al., 2018), and neurological diseases, e.g., multiple sclerosis (MS) and Alzheimer's disease (AD) (Reich et al., 2010; Klistorner et al., 2015; Mutlu et al., 2018; Wang et al., 2021). For different types of diseases, optic nerve fibers would represent varied symptoms like edema, demyelination, atrophy and degeneration at different locations along the visual pathway, which would change the original morphological and even structural characteristics of the optic nerve fibers. Also, the severity of the symptoms is highly relevant to disease progression. Dysfunction of optic nerve fibers would cause serious vision problems; describing the status of optic nerve fibers in morphology and structure can help determine a patient's condition and choose the appropriate treatment strategy.

In addition, it is not uncommon that optic nerve fibers get compressed or damaged due to tumors and traumas (Romano et al., 2007, 2009; Chamberland et al., 2018). It requires a clear delineation of the morphological and structural status of the optic nerve fibers to evaluate the damage. Meanwhile, the accurate locations of the optic nerve fibers in the brain play significant roles in neurosurgery for compression release and damage repair. The location information of the optic nerve fibers is also crucial for radiation therapy to protect the optic nerve fibers from radiation (Isambert et al., 2008; Dai et al., 2021).

Currently, there are several imaging techniques that can provide an in-vivo delineation of the optic nerve fiber in the visual pathway. Particularly, computed tomography (CT) and magnetic resonance imaging (MRI) are used to reveal the optic nerve fibers at the anterior visual pathway, i.e., from the optic disc to the LGN (Tamraz et al., 1999; Wichmann and Müller-Forell, 2004), while diffusion tensor imaging (DTI) is usually applied to delineate the optic nerve fibers at the OR (Dayan et al., 2015b). These imaging techniques make it possible to evaluate the morphological and structural status of the optic nerve fibers and target their locations in the brain via in-vivo approaches, and reconstructing the optic nerve fibers from medical images can further facilitate these approaches.

Manual optic nerve fiber reconstruction is difficult and time-consuming, thus computational approaches for automated optic nerve fiber reconstruction are developed. These computational approaches can be divided into two categories, i.e., image segmentation and fiber tracking. The former is used for CT/MRI images, while the latter is performed for DTI data. Despite the difference in implementation, these approaches face the same challenge, i.e., the thin-long structure of the optic nerve fibers. The thin-long structure makes the optic nerve fibers easily affected by the partial volume effect (PVE) (Mansoor et al., 2016). PVE can decrease the image contrast to neighboring tissues, increasing the difficulty of image segmentation (Cabezas et al., 2011); also, it enables multi-orientations in each voxel, raising the complexity of orientation estimation for fiber tracking (Alexander et al., 2001; Jeurissen et al., 2019). Though various computational approaches are proposed for this challenge in optic nerve fiber reconstruction, it has not been well addressed yet.

In recent years, some advanced techniques such as generative artificial intelligence (AI) have been developed and these techniques exhibit their potential in handling this challenge. Generative AI has demonstrated its power in image super-resolution and multi-modal image synthesis (Hu et al., 2020a,b, 2021; You et al., 2022). The major cause of PVE is the low image resolution, thus higher image resolution can help get it alleviated. Multi-modal image fusion is another way to resist PVE. Multi-modal images can provide consistent and complementary information to release the confusion caused by PVE. However, it is not common to see multi-modal approaches for optic nerve fiber reconstruction as the acquisition of multi-modal data would be expensive and time-consuming in clinical practice. Multi-modal image synthesis provides a cheap and efficient way to acquire multi-modal images (Hu et al., 2021), removing the biggest barrier that hinders multi-modal research on optic nerve fiber reconstruction.

To apply generative AI and other new techniques in optic nerve fiber reconstruction, it is better to gain a comprehensive understanding of the task and the existing methods. However, to the best of our knowledge, a comprehensive review of the computational approaches for the reconstruction of optic nerve fibers from medical images is still lacking. Therefore, we performed such a review in this paper. We started with the anatomy of the visual pathway and imaging techniques of the optic nerve fibers. Then, we described both the two strategies, i.e., image segmentation and fiber tracking, for optic nerve fiber reconstruction. For each strategy, both conventional and AI-based methods were introduced. Finally, we discussed the selection rules and future challenges to performing optic nerve fiber reconstruction, providing guidance for clinical application and future studies. More details can be viewed in the following sections.

## 2. Anatomy of visual pathway

**Figure 1:**
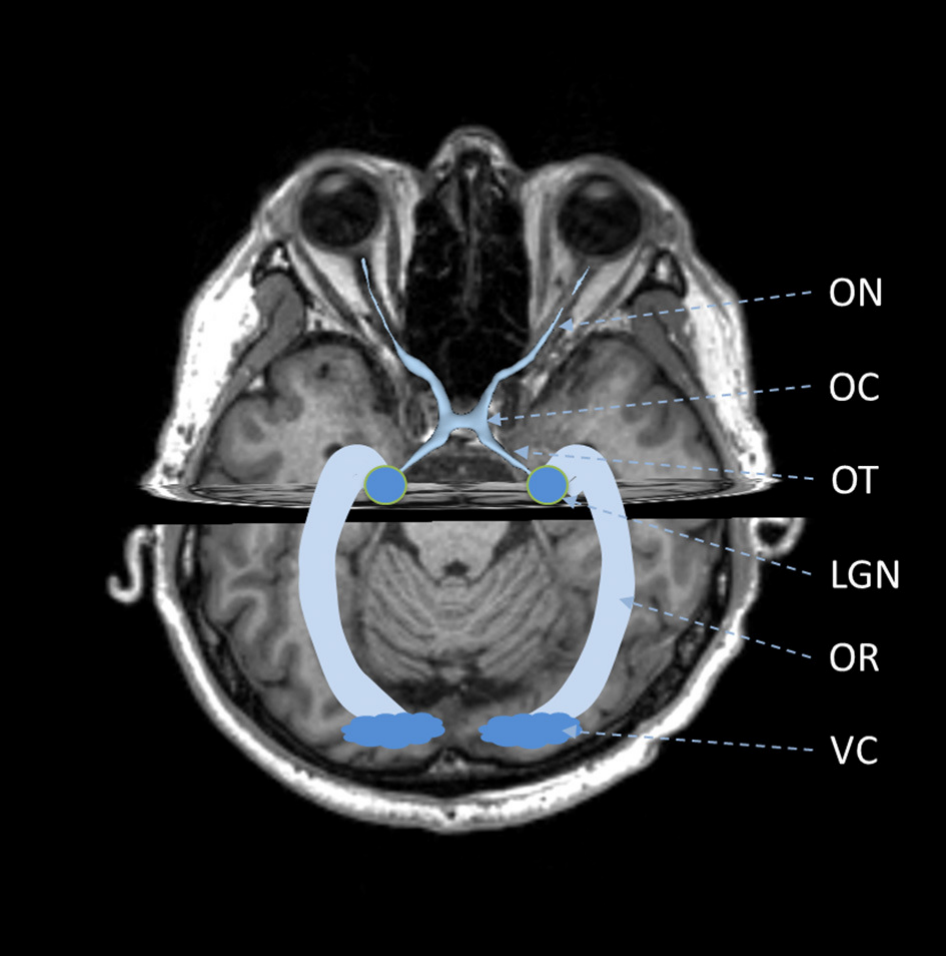
Schematic of anatomy of visual pathway. ON, optic nerve; OC, optic chiasm; OT, optic tract; LGN, lateral geniculate nucleus; OR, optic radiation; VC, visual cortex.

The visual pathway consists of the ON, OC, OT, LGN, OR, and VC (Tamraz et al., 1999; Smith and Strottmann, 2001; Wichmann and Müller-Forell, 2004; Jäger, 2005), as shown in Figure 1. The ON is the first part of the visual pathway. It is a thin-long myelinated fiber bundle formed by the axons of the retinal ganglion cells. There is a pair of ONs, which start from the optic disks of each eye, pass through the orbit and optic canals, and finally get crossed at the OC. Based on the locations, the ON can be further divided into four segments, i.e., the intraocular, intraorbital, intracanalicular, and intracranial segments. The lengths for the four segments are about 1 mm, 30 mm, 6 mm, and 10 mm, respectively. The OC is a flat x-shape structure located at the junction of the floor and the anterior wall of the third ventricle. It is just situated anteriorly to the pituitary stalk. In OC, only the optic nerve fibers from the medial retina (nasal side) would get crossed, while those from the lateral retina (temporal side) remain uncrossed. Then, the optic nerve fibers at each side keep going from the posterolateral angle of the OC and form the left and right OTs. The optic nerve fibers in OTs run backward and lateralward of the OC and wind around the midbrain. Most of these optic nerve fibers get terminated at the LGN, while there are also some passing over the LGN and reaching the superior colliculus to coordinate eye movements. The LGN is located in the lateral geniculate body, which is the posterior-inferior aspect of the thalamus. The LGN consists of alternating gray and white matter layers and serves as a relay station in the visual pathway (Fujita et al., 2001). The LGN projects the visual signal from the retina to the VC, and the optic nerve fiber connecting the LGN and VC form the optic radiation. The OR can be divided into three major fiber bundles, i.e., the dorsal, lateral, and ventral bundles. The dorsal and lateral bundles pass through the temporal and parietal lobes posteriorly and terminate at the occipital lobe; the ventral bundle runs anteriorly and laterally into the temporal lobe and bypasses the temporal horn of the lateral ventricle, generating the Meyer's loop (Tamraz et al., 1999; Dayan et al., 2015b). The VC is also called the striate cortex. It is located at the superior and inferior lips of the calcarine fissure.

It is seen that the major components of the visual pathway are optic nerve fibers. Separated by the LGN, the two types of optic nerve fibers share similar symmetric curved thin-long structures but vary from each other in length, diameter, and curvature. Also, they are located with different neighboring tissues. The optic nerve fibers in the anterior visual pathway, i.e., from the optic disc to the LGN, are mostly located around muscle, fat, cranium, and blood vessels, while those in the OR are next to the brain's gray and white matters. The differences in these anatomical characteristics lead to quite different representations in medical images. More details on imaging would be introduced in the next section.

## 3. Imaging of optic nerve fibers in visual pathway

Currently, CT, MRI and DTI are the common imaging techniques for in-vivo delineation of the optic nerve fibers in the visual pathway. CT and MRI images are usually used to assess the optic nerve fibers in the anterior visual pathway (Smith and Strottmann, 2001; Becker et al., 2010). In general, MRI is superior in imaging the optic nerve fibers than CT as it can achieve high contrast among soft tissues. In comparison with CT, it can more easily differentiate the optic nerve fibers from the complex adjacent tissues in the orbit and sellar regions. Also, MRI is free from radiation and is safer than CT. Nevertheless, CT has its advantage in revealing bony tissues and foreign bodies. It can reveal the damages to the visual pathway caused by orbital or optic canal trauma as well as the calcification of the optic nerve fibers due to the tumor compression. In addition, CT is less affected by motion artifacts and can be applied to people with metal implants, making it a better choice than MRI in some special clinical scenarios. Besides CT and MRI, DTI can also be applied to reconstruct the optic nerve fibers in the anterior visual pathway; and, it can provide more details such as the fiber crossing at the OC (He et al., 2021). But, DTI takes much longer scanning time than MRI and CT, making it less practical in clinical scenarios. Instead, DTI is more frequently applied to reconstruct the optic nerve fibers in the OR (Dayan et al., 2015a; Schurr et al., 2018). The three fiber bundles in OR are located very close to other white matter tracts; the subtle variations in white matter signal make it difficult to reveal the anatomical heterogeneity in OR from CT and conventional MR images (Yogarajah et al., 2009; Winston et al., 2012). DTI is a technique to monitor the motion of water molecules in the human body by collecting multi-gradient MRI images. As the motion of water molecules is bounded by the nerve fibers, its speed and direction can be used to describe the structure and orientation of nerve fibers. Unlike MRI and CT images, the structure of optic radiation cannot be directly viewed in raw DTI images. There is a need to calculate the DTI metrics or perform fiber tracking to reveal the structure of the OR. It is seen that the three imaging techniques have their unique advantages and their own application scenarios. Also, the different representations of the optic nerve fibers in images of different modalities require different reconstruction methods. Usually, image segmentation is applied to CT and MRI images where optic nerve fibers exhibit a certain image contrast to neighboring tissues, while fiber tracking is performed to DTI data to exploit the structural and orientational information for more precise delineation of the optic nerve fibers. Both image segmentation and fiber tracking approaches can be further classified as conventional and AI-based methods. In the following two sections, we would describe more details of both the conventional and AI-based methods using the two reconstruction strategies.

## 4. Fiber reconstruction by image segmentation

### 4.1. Conventional methods

Image segmentation is usually used to reconstruct optic nerve fibers from CT and MRI images. Conventionally, there are various methods to perform image segmentation, such as thresholding, boundary-based, region-based, model-based, atlas-based, etc (Despotović et al., 2015; Wang et al., 2016). The thresholding methods are not suitable for the segmentation of the optic nerve fibers given their poor image contrast with neighboring tissues at some segments of the visual pathway. Also, their thin-long structures make it difficult to perform boundary-based and region-based methods. It is found that most methods for optic nerve fiber reconstruction are model-based or atlas-based (Table 1).

**Table 1:** Conventional image segmentation methods for optic nerve fiber reconstruction.

**Method type**	**Method description**	**Anatomical region**	**Imaging modality**	**Research**
Model-based	Geometry model	ON	CT	Bekes et al., 2008
	Atlas-navigated optimal medial axis and deformable model (NOMAD)	ON, OC	CT, MRI	Noble and Dawant, 2011
	Weighted partitioned active shape model	ON, OC, OT	MRI	Yang et al., 2014
	PArtitioned Shape and Appearance Learning (PAScAL)	ON, OC, OT	MRI	Mansoor et al., 2015
Atlas-based	Single-atlas	ON	MRI	D'haese et al., 2003
	Multi-atlas, Post-processing	ON, OC	CT	Gensheimer et al., 2007
	Multi-altas	ON, OC	MRI	Isambert et al., 2008
	Multi-atlas, non-local model	ON	CT	Asman et al., 2013
	Multi-atlas, variable voxel resolution and field of view	ON	CT	Harrigan et al., 2014; Panda et al., 2014

ON, optic nerve; OC, optic chiasm; OT, optic tract.

Model-based methods would first define a model based on the prior information on the shape and appearance of the tissue to be segmented and then fit the model to the new images (Heimann and Meinzer, 2009). The models can be either fixed geometry models or deformable models. For the fixed geometry models, they can be easily fitted via an explicit parameter estimation based on selected landmarks. The deformable models such as active shape models, active appearance models and level-set are usually fitted with searching or optimization procedures. Particularly, Bekes et al. (2008) approximate the ON in a CT image as a cone and cylinder and fit the cone and cylinder using a semi-automatic way. This fixed-model-based approach is simple but its reproducibility is doubted. Noble and Dawant (2011) applied an atlas-navigated optimal medial axis and deformable model (NOMAD) to segment the ON and OC based on paired CT and T1-weighted MRI images. The exploitation of multi-modal images and hybrid methods (model- and atlas-based) enhances the segmentation results, but the paired CT and MRI images are not always available in clinical practice. Yang et al. (2014) proposed a weighted partitioned active shape model to segment the anterior visual pathway from T1-weighted MRI images. This method is also capable to segment the OT, which is believed as a more challenging task than ON and OC segmentation before this study. Mansoor et al. (2015) developed a method entitled PArtitioned Shape and Appearance Learning (PAScAL) to segment the anterior visual pathway from MRI images. This method can also be applied to the pathological anterior visual pathway.

Atlas-based methods treat the segmentation problem as a registration problem, i.e., aligning the new image and the atlas (Cabezas et al., 2011). Usually, an atlas contains two image volumes, one intensity image (template) and one segmented image (label). Image registration is used to build the geometrical connection between the new image and the template; then, the segmentation can be achieved by propagating the label to the image space via the geometrical connection. D'haese et al. (2003) manually drew an atlas that includes the ON based on visually selected MRI images and segmented the ON with the atlas. Gensheimer et al. (2007) extended single-atlas segmentation to multi-atlas segmentation and performed additional post-processing procedures including a ray casting algorithm, reshaping of unreasonable cross sections, and surface fitting to further modify the inaccurate contours. Isambert et al. (2008) applied a multi-atlas segmentation method to segment ON and OC from MRI images under clinical conditions. Asman et al. (2013) developed a non-local model to perform multi-atlas segmentation for the ON based on CT images. Harrigan et al. (2014) and Panda et al. (2014) paid attention to the robustness of the atlas-based segmentation for the ON and proposed an improved registration procedure.

### 4.2. AI-based methods

AI-based methods are data-driven approaches, which learn the rules from the data. Such approaches reduce manual operations like predefining models or atlases and are more easily implemented in practice. AI-based methods usually treat the segmentation procedure as a pixel/voxel-wise classification or clustering task. In the beginning, the classification/clustering is performed using conventional machine learning algorithms based on hand-crafted features. For example, Dolz et al. (2015) extracted features from neighborhood information and applied the support vector machine (SVM) to conduct the classification. With the occurrence and development of deep learning techniques, it becomes possible to integrate the feature extraction procedure into the learning process, further simplifying the procedure to segment the optic nerve fibers.

The studies on deep learning methods for optic nerve fiber segmentation from CT/MRI images have passed through three periods (Table 2). In the early period, deep learning methods are only used for feature extraction and segmentation is still implemented by conventional methods. For example, Mansoor et al. (2016) used a stacked auto-encoder to learn new feature representations for a model-based segmentation procedure. After this early period, deep learning is also used for pixel/voxel classification. At this stage, the network is usually formed by two network modules, e.g., a convolutional neural network (CNN) and a fully connected network, responsible for feature extraction and pixel/voxel classification, respectively. Based on this basic network structure, Ren et al. (2018) extended the original CNN to an interleaved structure for joint segmentation of optic nerve and chiasm; Dolz et al. (2017) replaced the CNN with a stacked denoised auto-encoders to learn a compact representation of the hand-crafted features; Duanmu et al. (2020) modified the CNN using a multi-resolution path approach to combine multi-scale features. Recently, a more powerful network, i.e., the U-Net, has been developed (Ronneberger et al., 2015). U-Net is composed of a down-sampling branch and an up-sampling branch. The down-sampling and up-sampling branches are made up of paired encoders and decoders, respectively. The down-sampling procedure can help extract the context information and the up-sampling procedure is used for fine localization. Also, there are skip connections between the encoders and decoders. As there might be information loss during the up-sampling procedure, the skip connections make it possible to combine the up-sampling results with the original information before the down-sampling procedure. With the skip connections, the localization can be more accurate. Compared with the two-module network, U-Net further integrates the feature extraction and pixel/voxel classification procedures.

**Table 2:** AI-based image segmentation methods for optic nerve fiber reconstruction.

**Method type**	**Method description**	**Anatomical region**	**Imaging modality**	**Dataset**	**Research**
Machine Learning	SVM	ON	MRI	Private	Dolz et al., 2015
CNN only	Stacked auto-encoder	ON, OC, OT	MRI	Private	Mansoor et al., 2016
CNN+FCN	Stacked denoised auto-encoders+FCN	ON, OC	MRI	Private	Dolz et al., 2017
	Interleaved CNN+FCN	ON, OC	CT	PDCCA	Ren et al., 2018
	Multi-resolution multi-scale CNN+FCN	ON, OC	CT	Private	Duanmu et al., 2020
U-Net-Like	Squeeze-excitation Block	ON, OC	CT	PDDCA, TCIA	Zhu et al., 2019
	Connection Enhancement, Global restriction	ON, OC	CT	PDCCA	Tong et al., 2018
	Connection Enhancement, Global restriction	ON, OC	CT	PDCCA	Tong et al., 2019
	Connection Enhancement	ON, OC	CT	Private	Zhu et al., 2021
	Recursive ensemble segmentation	ON, OC	MRI	Private	Chen et al., 2019
	Localization+Segmentation	ON, OC	CT	PDCCA	Wang et al., 2019
	Localization+Segmentation, Atlas information	ON, OC, OT	MRI	Private	Zhao et al., 2019
	Localization+Segmentation, Atlas information	ON, OC, OT	CT, MRI	Private, PDCCA	Ai et al., 2020
	Localization+Segmentation	ON, OC	MRI	Private	Liu and Gu, 2020
	Localization+Segmentation	ON, OC	CT	PDCCA	Amjad et al., 2022
	Pre-processing	OC	MRI	CHIAS M	Puzniak et al., 2021b
	Pre-processing	ON	CT	TCIA	Ranjbarzadeh et al., 2022
	Post-processing	ON, OC	CT	Private	Ibragimov and Xing, 2017
	Post-processing	ON, OC	MRI	Private	Mlynarski et al., 2020

ON, optic nerve; OC, optic chiasm; OT, optic tract; SVM, support vector machine; CNN, convolutional neural networks.

The state-of-the-art (SOTA) methods for optic nerve fiber reconstruction from CT/MRI images are mostly based on the U-Net. Particularly, some researchers tried to modify the encoders and decoders as well as their connections to enhance context information exploitation. For example, Zhu et al. (2019) added squeeze-excitation blocks into the down-sampling and up-sampling approaches; Tong et al. (2019) and Zhu et al. (2021) tried DenseNet and V-Net, which enhance the connections among encoders and decoders, to segment the ON and OC from CT and MRI images. Also, some researchers tried to add global loss restrictions to avoid irregular segmentation results due to the pixel/voxel-wise segmentation strategy. Specifically, Tong et al. (2018, 2019) added a latent shape restriction as well as an adversarial restriction to guarantee the global shape of the segmented ON and OC. Besides the modification of network blocks and losses, some researchers paid attention to the training strategies. Chen et al. proposed a recursive ensemble organ segmentation framework. In this framework, the organs that are easily segmented, e.g., the eyeballs, would be first segmented; and then, the segmentation results are fed to the network together with the original inputs for the segmentation of more complicated organs like ON and OC (Chen et al., 2019). Wang et al. proposed a hybrid network containing two U-Nets for localization and segmentation, respectively. The U-Net for localization was named “LocNet” and used to localize the region of the ON, while the one for segmentation was named “SegNet” and applied only in the extracted region to exclude other interference (Wang et al., 2019). Zhao et al. adopted a similar strategy but replaced the LocNet with an atlas-based approach, i.e., performing registration between the atlas and a new image to localize the ON. They also generated a spatial probabilistic distribution map using the atlas to assist the segmentation (Zhao et al., 2019; Ai et al., 2020). Differently, Liu and Gu (2020) and Amjad et al. (2022) replaced the SegNet with a two-module network, where the CNN adopted a multi-resolution structure.

In addition to the deep learning networks, researchers also tried to enhance the segmentation results using proper pre-processing and post-processing approaches. For the pre-processing, Puzniak et al. (2021b) applied a data-augmentation strategy to train a 3D U-Net. Ranjbarzadeh et al. (2022) pre-processed the input images by combining a fuzzy C-mean clustering algorithm, histogram equalization, and a texture descriptor based on the local directional number. For post-processing, Ibragimov and Xing (2017) proposed a post-processing procedure based on Markov random fields. Mlynarski et al. (2020) developed a graph-based post-processing approach to guarantee the connectivity between the eyes and OC.

## 5. Fiber reconstruction by fiber tracking

### 5.1. Conventional methods

Fiber tracking, also called fiber tractography, is a computational procedure to reconstruct nerve fibers from DTI images. Although there is a debate on the reliability of fiber tracking in delineating the true brain nerve fibers, it has been widely applied in both medical research and clinical practice. There are also plenty of studies focusing on the reconstruction of optic nerve fibers, especially for the OR, using fiber tracking.

Fiber tracking would estimate a series of streamlines to delineate the global fiber tractography using deterministic, probabilistic, or global algorithms (Jeurissen et al., 2019; Li et al., 2020). Deterministic algorithms are proposed based on the assumption that there is a predominant orientation in each voxel of DTI images. Common deterministic algorithms include streamlines tracking (STT) (Basser, 1998; Basser et al., 2000), fiber assignment by continuous tracking (FACT) (Mori et al., 1999; Chao et al., 2008), Tensor-lines (Weinstein et al., 1999), tensor deflection (TEND) (Lazar et al., 2003), and vector criterion tracking (VCT) (Kim et al., 2004). These algorithms usually select the diffusion tensor as the model to describe fibers' microstructures at each voxel. But, the diffusion signal would be inevitably distorted by noise and artifacts, affecting the certainty of voxel orientation inferred from the diffusion tensor (Jones, 2010). The assumption of one orientation per voxel is also doubted due to the existence of crossing fibers (Behrens et al., 2007). The existence of these problems raises concerns about the deterministic algorithms; the probabilistic algorithms are then proposed. To cope with the uncertainty, the probabilistic algorithms use the probability density functions (PDF) (Behrens et al., 2003) and fiber orientation distribution (FOD) (Tournier et al., 2004) to represent fibers' microstructures at each voxel. Based on these probabilistic models, the algorithms like probabilistic index of connectivity (PICo) (Parker et al., 2003), unscented Kalman filter (UKF) (Malcolm et al., 2010), probabilistic tracking with crossing fibers (PROBTRACKX) (Behrens et al., 2007), ConTrack (Sherbondy et al., 2008), particle filtering tractography (PFT) (Zhang et al., 2009), and 2nd-order Integration over Fiber Orientation Distributions (iFOD2) (Smith et al., 2012) are proposed. Compared with deterministic algorithms, probabilistic algorithms can delineate more complicated nerve fiber distributions; but, they would also cause a large number of false positive streamlines and suffer from heavy computational costs. Both the deterministic and probabilistic algorithms are based on local information, while global algorithms treat fiber tracking as a global optimization problem. The existing global algorithms can be mostly divided into two categories, i.e., graph-based algorithms (Iturria-Medina et al., 2007) and Gibbs algorithms. Graph-based algorithms should set the seeding and targeting regions, which is not necessary for Gibbs algorithms (Kreher et al., 2008). Global algorithms can avoid the error accumulation problem in local algorithms and reduce the number of false positive streamlines; but, their computational costs are much greater than local algorithms and convergent solutions are not guaranteed.

Besides the algorithm, there are also some key operations and settings to ensure an accurate fiber tracking procedure (Jacquesson et al., 2019; Jeurissen et al., 2019). For local algorithms and graph-based global algorithms, there is a need to determine the seeding and target regions of interest (ROIs). The seeding and target ROIs mean the two ends of the generated fibers by the tracking algorithms. Except for the whole-brain tracking, these two ROIs can be drawn in a manual way (Rossi-Espagnet et al., 2020; Haykal et al., 2022) or by projecting the labels in a built brain atlas (Karahan et al., 2019; Papadopoulou et al., 2021). In addition, the ROIs can also be acquired by other fiber tracking procedures (Davion et al., 2020). Besides these two types of ROIs, there are also inclusive and exclusive ROIs for the filtering of valid fibers (Horbruegger et al., 2019). In addition, some thresholds to constrain the fibers' lengths, curvatures/angles, and fractional anisotropy (FA)/fiber orientation distribution function (fODF) values are also set for the filtering process.

The specific methods for optic nerve fiber reconstruction are shown in Table 3. The reconstruction of the optic nerve fibers from DTI images follows the above fiber tracking frameworks; but, the selection of tracking algorithms, ROI drawing, and thresholds setting would change with the location of optic nerve fibers. Particularly, deterministic algorithms can be applied to the optic nerve fibers in the anterior visual pathway (Dasenbrock et al., 2011; De Blank et al., 2013; Takemura et al., 2017; Hofstetter et al., 2019; Jin et al., 2019) but they are not suggested for OR reconstruction (Yogarajah et al., 2009). The OR region is close to the neighboring white matter tracts and image voxels in this region are more likely to contain multiple orientations. The probabilistic algorithms can be applied for both the two types of optic nerve fibers (Dayan et al., 2015a; Kammen et al., 2016; Zolal et al., 2016; Backner et al., 2018; Yoshimine et al., 2018; Ather et al., 2019; Glick-Shames et al., 2019; Wu et al., 2019; Davion et al., 2020; Lacerda et al., 2020; Rossi-Espagnet et al., 2020; Reid et al., 2021; Haykal et al., 2022; Liu et al., 2022); but, there are still some differences. The probabilistic algorithms are proposed to handle the uncertainty and they can be classified into different categories based on the source of the uncertainty (Jeurissen et al., 2019). The reconstruction of optic nerve fibers at the anterior visual pathway and in the OR has different uncertainty sources. The former's uncertainty comes from the interference of the complicated skull base environment, which contains nerves, bone, air, soft tissue, and cerebrospinal fluid; the latter's uncertainty is mainly due to the multi-orientation problem. The difference in uncertainty sources would affect the selection of the probabilistic algorithms. In addition, the seeding ROIs for the ON and OT reconstruction are usually set as the end of the eyeballs and the OC, respectively, while those for the OR are set as the LGN. The target ROIs include the OC, LGN and VC for the reconstruction of the ON, OT and OR, respectively. The other settings like the inclusive and exclusive ROIs as well as the thresholds would be more task-specific.

**Table 3:** Conventional fiber tracking approaches for optic nerve fiber reconstruction.

**Method type**	**Model/algorithm**	**Anatomical region**	**Software**	**Research**
Deterministic	Tensor/FACT	OT	DTI Studio	Dasenbrock et al., 2011
	Tensor/FACT	OT	TrackVis	Jin et al., 2019
	Tensor/FACT	OT	dTV II FZRx	Takemura et al., 2017
	Tensor/FACT	ON, OT, OR	DTI Studio	De Blank et al., 2013
	FOD/STT	OT, OR	ExploreDTI	Hofstetter et al., 2019
Probabilistic	Tensor/PICo	ON	FSL	Zolal et al., 2016
	Tensor/PICo	OR	Camino	Dayan et al., 2015a
	Tensor/ConTrack	OT, OR	VISTA	Backner et al., 2018
	Tensor/ConTrack	OT, OR	VISTA	Glick-Shames et al., 2019
	Tensor/ConTrack	OT, OR	VISTA	Yoshimine et al., 2018
	Tensor/PROBTRACKX	ON, OT	FSL	Wu et al., 2019
	Tensor/PROBTRACKX2	ON, OC, OT	FSL	Ather et al., 2019
	FOD/iFOD2	OR	MRtrix3	Davion et al., 2020
	FOD/iFOD2	OR	MRtrix3	Lacerda et al., 2020
	FOD/iFOD2	OT, OR	MRtrix3	Rossi-Espagnet et al., 2020
	FOD/iFOD2	OR	MRtrix3	Reid et al., 2021
	FOD/iFOD2	OT, OR	MRtrix3	Haykal et al., 2022
	FOD/iFOD2	OR	MRtrix3	Liu et al., 2022

ON, optic nerve; OC, optic chiasm; OT, optic tract; OR, optic radiation; FOD, fiber orientation distribution.

### 5.2. AI-based methods

The conventional framework for fiber tracking is a complicated procedure containing the processes like pre-processing, seeding, tractography, and filtering of valid streamlines. Although several softwares integrate these processes (Table 3), the operations like ROI drawing, tracking algorithm selection, and threshold setting still require manual implementation. In recent years, AI technique has developed rapidly; researchers are trying to replace these manual operations with automated ways using AI technique (Table 4).

**Table 4:** AI-based fiber tracking approaches.

**Process**	**AI model**	**Research**
Pre-processing	CNN	Tian et al., 2020
	DNN	Koppers et al., 2017
	U-Net-like	Jha et al., 2022a
	SRN	Zeng et al., 2022
	Encoder-decoder, Discriminator	Jha et al., 2022b
	SuperDTI network	Li et al., 2021
	MLP, U-Net	Karimi et al., 2021a,b,c
Seeding	U-Net	Avital et al., 2019
	U-Net-like	Wasserthal et al., 2019
Tractography	Random forest classifier	Neher et al., 2017
	RNN	Poulin et al., 2017, 2018
	MLP	Jörgens et al., 2018;
	MLP	Wegmayr et al., 2018
	Reinforcement learning	Théberge et al., 2021
	U-Net-like	Wasserthal et al., 2018
Filtering	U-Net-like	He et al., 2023
	CNN	Xu et al., 2019
	CNN	Zhang et al., 2020
	Siamese networks	Chen et al., 2021
	Contrast learning	Xue et al., 2022, 2023

CNN, convolutional neural networks; DNN, deep neural networks; RNN, recurrent neural networks; SRN, super-resolution networks.

AI-based methods are applied first in the tractography process. Neher et al. (2017) tried to perform the tractography by machine learning. They applied a random forest classifier to learn multiple potential directions of a streamline from the raw diffusion signals and determined the streamline's progressing direction and termination using a neighborhood sampling strategy and a voting scheme, respectively. Poulin et al. treated the tractography as a regression problem and proposed the recurrent neural networks (RNN) to acquire the mapping between the diffusion signal and the streamlines' directions for both whole-brain and bundle-specific tractography (Poulin et al., 2017, 2018). The RNN can exploit both the new observations and the past seen information along the tracked streamlines. In addition to the diffusion signals, Jörgens et al. (2018) further pointed out the importance of the previous step directions for the tractography. They adopted an alternative way to predict the next step direction of a streamline via a multi-layer perceptron (MLP), whose input is a vector acquired by concatenating the diffusion signals and previous step directions. Wegmayr et al. (2018) also used an MLP to perform the tractography and further validated the significance of previous step directions; but, they changed the input of the MLP as a vector formed by a flattened data block and several incoming vectors. The tractography can also be implemented via reinforcement learning and Théberge et al. (2021) proposed a general framework for this strategy. Apart from these local tractography methods, Wasserthal et al. (2018) developed a U-Net-like network to directly reconstruct the fiber tracts from the fields of fODF peaks.

Recently, several AI-based methods have been applied in processes other than tractography. For the pre-processing approach, AI-based methods focus on two aspects, i.e., generating high-fidelity diffusion signals from low-quality input and building the diffusion model from the raw diffusion signals. Acquiring high-fidelity diffusion signals usually requires a certain number of diffusion-encoding directions and multi-shell acquisitions, which takes a long scanning time. Tian et al. (2020) proposed a 10-layer CNN to reduce the requirement on the number of diffusion-encoding directions, particularly limiting the number to the minimum level for diffusion tensor calculation. Koppers et al. (2017) and Jha et al. (2022a) reconstructed the multi-shell diffusion signals from single-shell acquisitions using DNN and U-Net-like network, respectively. Zeng et al. (2022) proposed a super-resolution network to enhance the FOD model that was built based on the single-shell acquisition, and Jha et al. (2022b) developed a more complicated network containing multiple encoder-decoder structures and discriminators. Mapping raw diffusion signals to diffusion models is also very challenging. It is quite difficult for conventional methods to estimate the fibers' number and orientations per voxel from raw diffusion signals. Li et al. (2021) demonstrated the advantages of AI-based methods in this challenging task. They proposed a SuperDTI network for diffusion model generation and the test results suggest that their model is less sensitive to noise and more robust to misregistration than conventional tensor fitting methods. Karimi et al. (2021a,b,c) further verified the superiority of AI-based methods via a series of explorations on diffusion metric map generation, fODF generation and fibers' number and orientations estimation. In addition to the pre-processing process, AI-based methods are also used to achieve automatic seeding. Avital et al. (2019) and Wasserthal et al. (2019) tried automated seeding using U-Net and U-Net-like network, respectively. There are also studies focusing on AI-based automated filtering of valid streamlines. Particularly, AI can be used to draw inclusive or exclusive ROIs, such as He's work (He et al., 2023). Also, AI models can be used to directly classify or cluster the reconstructed streamlines (Xu et al., 2019; Zhang et al., 2020; Chen et al., 2021; Xue et al., 2022, 2023).

Although multiple AI-based methods are proposed for fiber tracking, the application of these methods in optic nerve fiber reconstruction is still rare. To the best of our knowledge, Reid et al. (2021) applied a U-Net-like network to automatically draw seeding ROI at the optic tract. He et al. (2023) proposed a unified global tractography framework for automatic visual pathway reconstruction. Li et al. (2022) used a modified SupWMA network to cluster the streamlines in the anterior visual pathways. These methods demonstrate the feasibility and effectiveness of AI-based methods in optic nerve fiber reconstruction, while there is still room for further improvement.

## 6. Discussion

Optic nerve fiber reconstruction is a common step to evaluate or project optic nerve fibers in clinical diagnosis and treatment. As shown in section 2, optic nerve fibers have thin-long structures and varying curvatures at different segments of the visual pathway, making them difficult to evaluate in either qualitative or quantitative ways without the reconstruction from the medical images. Also, manual delineation of the optic nerve fibers would be a tough task and costs a lot of time. As a result, computational methods are highly needed for clinical applications on optic nerve fibers. It is found that optic nerve fibers can be revealed in images of multiple modalities and there are different reconstruction strategies for each imaging modality. Also, each reconstruction strategy has both conventional and AI-based implementations. This paper reviews the existing computational methods to guide optic nerve fiber reconstruction in medical research and clinical practice and demonstrates the trend for future studies.

CT and MRI images are widely used for the visualization of the optic nerve fibers at the anterior visual pathway, i.e., from the end of the eyeballs to the LGN, while DTI can be used to visualize the optic nerve fibers along the entire visual pathway. Even though, DTI would not replace CT and MRI for optic nerve fiber reconstruction in clinical practice at the current stage. On one hand, DTI is with longer scanning time and lower image resolution than CT and MRI, making it less applicable in clinical practice. On the other hand, there are still debates on the consistency between the reconstructed fibers from DTI data and the real fibers in anatomy (Jeurissen et al., 2019), which limits its application scenarios such as the OAR drawing in radiation therapy.

Image segmentation and fiber tracking are two different reconstruction approaches for CT/MRI and DTI, respectively. Besides that, there are some other differences between these two approaches. Fiber tracking can achieve a more precise delineation of the optic nerve fibers than image segmentation, allowing the extraction of more accurate features to describe the morphological and structural changes of optic nerve fibers. For example, optic nerve fiber degeneration can be described by the volume change based on image segmentation results while it can be more precisely evaluated by the reduction in optic nerve fiber number based on fiber tracking results. Nevertheless, fiber tracking is time-consuming and its computational process is complicated and easily affected by noises and artifacts (Tournier et al., 2002; Lazar and Alexander, 2003). Also, it is not uncommon that there are false positive results and it requires abundant experience and enough knowledge of brain anatomy to ensure an accurate result (Jeurissen et al., 2019). These drawbacks restrict the scenarios where it can apply in clinical practice. In comparison to fiber tracking, image segmentation would be more efficient and robust; also, its results can be easily evaluated.

In comparison with conventional methods, AI-based methods are believed to be the trend for both image segmentation and fiber tracking. For image segmentation, AI-based methods are preferred to conventional model-based and atlas-based methods. Model-based methods require the design of complicated models to fit the thin-long structure of the optic nerve fiber; such models are difficult to estimate based on the complex background along the visual pathway and their robustness is doubted. Atlas-based methods require the registration between the target and template, while it is not easy to get two images fully aligned given the individual differences and interference from noises and artifacts. AI-based methods are data-driven approaches, which can automatically learn rules from complicated data. AI-based methods are more easily performed than those conventional methods and demonstrate much better segmentation accuracy and robustness. The only disadvantage of AI-based methods now is their high demand for fine-annotated labels.

For fiber tracking, the superiority of AI-based methods over conventional methods is not as great as image segmentation at the current stage. On one hand, AI-based methods are mostly proposed for one certain step of the fiber tracking procedure and a proper end-to-end AI-based fiber tracking approach is still lacking. On the other hand, the conventional methods for each fiber tracking step have been well integrated into toolboxes and software, decreasing their difficulty in implementation. Even though, it is seen that more and more studies on fiber tracking are trying to replace the conventional methods with AI-based ones.

It is also noticed that there are still some challenges in the reconstruction of optic nerve fibers from medical images with AI-based methods. These challenges point out the direction of future studies. The first challenge is the thin-long structure of the optic nerve fibers. The long optic nerve fibers pass through various brain regions that are formed by different brain tissues, yielding complicated contextual information. Meanwhile, the thin structure makes the signal intensities of the optic nerve fibers easily affected by their neighboring tissues due to the PVE, yielding varied signal intensities at different segments of the visual pathway. The existing image segmentation methods applied multi-scale, coarse-to-fine, or iterative strategies to handle the variations in signal intensity and contextual information; pre-processing and post-processing are also used to modify the false-positive and missing voxels. Even though measures are taken, it is seen that the improvement is far from satisfactory, suggesting that the current local voxel-based segmentation strategy would not be powerful enough to handle such a complicated problem. Also, the long optic nerve fibers have varied curvatures. In existing fiber tracking frameworks, the curvature is a significant sign for tracking termination and fiber selection. The varied curvatures increase the difficulty of setting these rules. Furthermore, it requires a large field of view to reveal the long optic nerve fibers in an image at the current stage. To achieve such a field of view, the image resolution has to be sacrificed to maintain an acceptable scanning time in clinical practice, increasing the PVE. Thus, more powerful segmentation and fiber tracking strategies are required to cope with the challenges brought by the thin-long structure.

The second challenge is the lacking of task-specific datasets. AI-based methods are data-driven methods and their performance highly depends on the quality of data. To the best of our knowledge, most existing studies on image segmentation and fiber tracking are based on private datasets, which are not available to the public. There are a minor number of publicly available datasets, such as PDDCA (Raudaschl et al., 2017), TCIA (Clark et al., 2013; Zhu et al., 2019), and CHIASM (Puzniak et al., 2021a,b) for image segmentation and HCP for fiber tracking. However, these datasets are not initially collected for optic nerve fiber reconstruction. Most of these datasets require further cleaning and annotation operations. Meanwhile, the imaging protocols and pre-processing steps in these datasets may not be consistent with those used in clinical practice. Also, in some of the datasets, the images only cover part of the optic nerve fibers and cannot be used to reconstruct the entire visual pathway. In addition, the situations like multi-modal images and disease-specific deformation are not fully considered in these existing datasets. Therefore, building a dataset specifically for optic nerve fiber reconstruction is in great need.

The third challenge is the control of computational cost. For image segmentation, more powerful segmentation networks are usually with more complicated network structures at the current stage. Also, the inputs are 3D brain images for the reconstruction of optic nerve fibers. These together indicate a high computational cost. For fiber tracking, the tractography is usually an iterative process and time-consuming for both conventional and AI-based methods. The high computational cost would reduce the value of clinical application. The way to balance the computational cost and reconstruction performance would be another challenge in future studies.

There are some new techniques such as generative AI that can help address these challenges. Generative AI has demonstrated its power in image super-resolution and image synthesis. Image super-resolution can be used to cope with the low-resolution problem caused by the large field of view. Also, image synthesis can be used to generate more data to get full exploitation of the existing datasets. In addition, it is realized that multi-modal fusion would be a possible way to enhance the performance of optic nerve fiber reconstruction. There are many other examples to support its effect on segmentation (Menze et al., 2014; Ibtehaz and Rahman, 2020; Wang et al., 2022). The combination of segmentation results and fiber tracking has once been explored (Reid et al., 2021; He et al., 2023). The segmentation results can be used as the seeds for tractography or the masks to filter valid streamlines. Therefore, developing new fusion and combination methods would be a feasible way to improve the reconstruction performance. Nevertheless, this kind of method would face the problem that multi-modal images are difficult to acquire in clinical practice. Generative AI provides a way for multi-modal image synthesis. Thus, in the future, we can try these new techniques in optic nerve fiber reconstruction.

## 7. Conclusion

In this paper, we provided a comprehensive review of the current SOTA computational methods for the reconstruction of optic nerve fibers. We described the difficulties to delineate or evaluate the optic nerve fibers directly from medical images, suggesting the necessity of optic nerve fiber reconstruction. We reviewed both the image segmentation and fiber tracking methods and the successful application of these methods in previous studies indicates the feasibility and effectiveness of computational methods in optic nerve fiber reconstruction. Also, we introduced both the conventional and AI-based implementations, and there is no doubt that AI-based methods are better choices for optic nerve fiber reconstruction. Meanwhile, we also pointed out the challenges for the existing AI methods, and future studies are needed to address these challenges.

## Author contributions

RJ and JL contributed to conception and design of the reviewing process. YC, SZ, TY, and HF searched and sorted the literatures. HJ, YH, and XZ provided key suggestions on the anatomy of visual pathway, imaging techniques, and image segmentation methods. RJ wrote the first draft of the manuscript. All authors contributed to manuscript revision, read, and approved the submitted version.
